# Glycoconjugates and Related Molecules in Human Vascular Endothelial Cells

**DOI:** 10.1155/2013/963596

**Published:** 2013-09-19

**Authors:** Norihiko Sasaki, Masashi Toyoda

**Affiliations:** Research Team for Geriatric Medicine (Vascular Medicine), Tokyo Metropolitan Institute of Gerontology, Sakaecho 35-2, Itabashi-ku, Tokyo 173-0015, Japan

## Abstract

Vascular endothelial cells (ECs) form the inner lining of blood vessels. They are critically involved in many physiological functions, including control of vasomotor tone, blood cell trafficking, hemostatic balance, permeability, proliferation, survival, and immunity. It is considered that impairment of EC functions leads to the development of vascular diseases. The carbohydrate antigens carried by glycoconjugates (e.g., glycoproteins, glycosphingolipids, and proteoglycans) mainly present on the cell surface serve not only as marker molecules but also as functional molecules. Recent studies have revealed that the carbohydrate composition of the EC surface is critical for these cells to perform their physiological functions. In this paper, we consider the expression and functional roles of endogenous glycoconjugates and related molecules (galectins and glycan-degrading enzymes) in human ECs.

## 1. Introduction

 Vascular endothelial cells (ECs) constitute the inner lining (endothelium) of blood vessels that form an interface between the blood and the vessel wall. Blood vessels alter their morphology and function in response to changes in blood flow, and their responses are based on blood flow detection by the vascular endothelium. ECs sense shear stress generated by flowing blood and transmit the signal to the interior of the cell, thereby evoking a cellular response [1]. The EC response to shear stress is closely linked to the regulation of vascular tone, blood coagulation and fibrinolysis, angiogenesis, and vascular remodeling. ECs also control vascular barrier regulation, passive diffusion, and active transport of substances from the blood [2]. Thus, ECs play important roles in vascular homeostatic functions, and excess activation or dysfunction of ECs is considered to lead to the development of vascular-related diseases, such as restenosis, arteriosclerosis, and cancer. 

 Carbohydrate antigens (also called glycans) are expressed on the cell surface as components of glycoproteins, glycosphingolipids, and proteoglycans; these carbohydrate antigens contribute significantly to fundamental biological functions, such as cell differentiation, cell adhesion, cell-cell interaction, pathogen-host recognition, toxin-receptor interactions, cancer metastasis, immune responses, and regulation of signaling pathways [3]. Several studies have revealed that glycoconjugates play key roles in vascular biology. In this paper, we describe the importance of glycoconjugates in human ECs, with respect to their regulated expression and functional roles, particularly under pathological conditions. 

## 2. Glycoproteins

 Glycoproteins are proteins that contain oligosaccharide chains (glycans) covalently attached to polypeptide side chains. The carbohydrate is attached to the protein as a cotranslational or posttranslational modification. This glycosylation of proteins is important for their physicochemical and biological properties, such as protein folding, stability, targeting, dynamics, and ligand binding [3]. 

**Figure 1:**
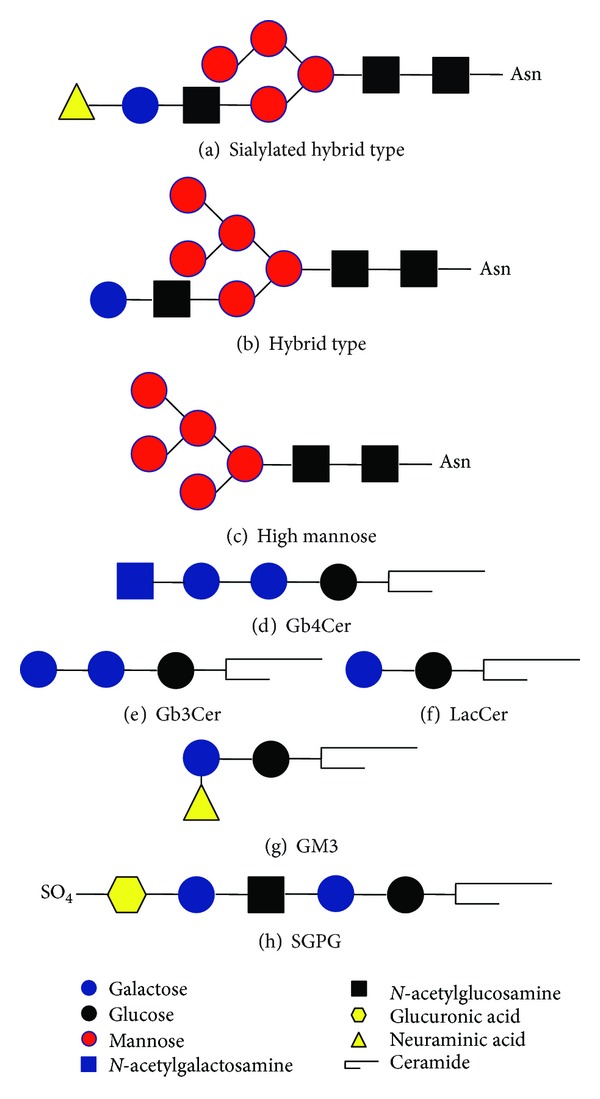
Structures of glycoconjugates. (a) One of the sialylated hybrid-type glycans described in reference [4] is shown. (b) and (c) *N*-glycans described in reference [5] are shown. (d)–(h) GSLs described in each reference are shown.

The vascular endothelial cadherin (VE-cadherin), including seven potential N-glycosylation sites, is endothelium specific and belongs to endothelial adherens junctions. Human VE-cadherin was purified from cultured human umbilical cord vein endothelial cells (HUVECs), and its glycosylation pattern was analyzed in order to enable further functional investigations [4]. The results revealed that VE-cadherin carries predominantly sialylated diantennary and hybrid-type glycans in addition to some triantennary and high-mannose-type species (sialylated hybrid type is shown in Figure 1(a)). Sialidase treatment of whole cells to remove cell surface sialic acids changed VE-cadherin immunofluorescence from a continuous netlike superstructural organization to an unevenly scattered one. These results indicate that cell surface sialic acids might play a role in VE-cadherin organization.

To date, several studies have been performed to unravel the relationship between changes in the expression of carbohydrate structures and the function of ECs in pathological conditions. TNF-*α* is one of the inflammatory factors that activate endothelial cells at sites of injury to recruit immune cells in acute and chronic inflammatory processes. Treatment with TNF-*α* differentially modulates a set of glycosylation-related genes, resulting in the increase of the cell membrane-associated glycans, such as *α*-2,6-sialic acid and fucose-*α*-1,2-galactose-*β*-1,4*-N*-acetylglucosamine [6]. Also, TNF-*α* stimulates endothelial expression of *N*-glycans, specifically the high-mannose and/or the hybrid types (Figures 1(b) and 1(c)), at cell junctions, and these epitopes play a role in modulating monocyte rolling and adhesion to the endothelium [5]. 

 In pathological conditions such as tumorigenesis and atherogenesis, endothelial dysfunction may be related to a change in cell surface glycans as follows. Using a flow cytometry assay with glycan-specific lectins, the expression profiles of a selected group of 9 carbohydrate structures (*β*-1,6-GlcNAc branching, high-mannose *N*-glycans, *α*-linked fucose residues, *N*-acetylglucosamine, *α*-2,3-sialic acid, *N*-acetyllactosamine, *α*-2,6-sialic acid, *α*-mannose, and *β*-1,4-galactose) were compared in HUVECs under control and tumor-conditioned medium- (TCM-) treated conditions. The expression of 6 of these structures (*β*-1,6-GlcNAc branching, high-mannose *N*-glycans, *N*-acetyllactosamine, *α*-2,6-sialic acid, *α*-mannose, and *β*-1,4-galactose) increased significantly after TCM treatment. In particular, the *β*-1,6-GlcNAc branching glycan expression level was greatly elevated after the stimulation [7]. This *β*-1,6-GlcNAc branching glycan was demonstrated to initiate endothelial cell contraction and gap formation, and these events lead to subsequent biological events such as tumorigenesis. High-mannose-type *N*-glycans increase in HAECs exposed to oscillatory shear or TNF-*α* [8]. Increasing surface N-linked mannose by inhibiting *N*-glycan processing potentiated monocyte adhesion under flow during TNF-*α* stimulation. Conversely, enzymatic removal of high-mannose *N*-glycans or masking mannose residues with lectins significantly decreased monocyte adhesion under flow. These results, therefore, indicate that surface N-linked mannose on ECs is a novel ligand for monocyte adhesion during atherogenesis.

## 3. Glycosphingolipids

A glycosphingolipid (GSL) is composed of a glycan structure attached to a lipid tail containing the sphingolipid ceramide. GSLs are widely expressed on cell membranes in lower and higher eukaryotic organisms. GSLs have frequently been used as important developmental marker molecules and have been suggested to have important biological functions [3, 9].

 The expression profile of GSLs in HUVECs was investigated under normal conditions [10] and after activation by inflammatory stimuli [11]. Inflammatory cytokines, such as IFN-*γ* and IL-1, have been known to alter expression of cell surface molecules in ECs. IFN-*γ* has a striking effect on the surface expression of GSLs, in particular on the surface expression of the major neutral GSL, globoside (globotetraosylceramide, Gb4Cer) (Figure 1(d)), but IFN-*γ* does not alter the total quantity of GSLs. By contrast, IL-1 increases the cell content of neutral and acidic GSLs but does not alter their surface expression. 

 ECs are believed to play an important role in the pathogenesis of hemolytic uremic syndrome (HUS). EC damage by *Escherichia coli* verocytotoxin *in vitro* is potentiated by additional exposure to inflammatory mediators such as TNF-*α*. The inflammatory mediators TNF-*α* and IL-1 make EC sensitive to verotoxin by elevation of verotoxin receptors such as GSL globotriaosylceramide (Gb3Cer/CD77) (Figure 1(e)) on the cell surface [12, 13]. ECs demonstrated that the level of EC sensitivity to verotoxin depends on the expression of Gb4Cer synthase [14].

 Functional analysis of GSLs on human ECs in angiogenesis has been performed. The mechanism of VEGF-driven angiogenesis involving lactosylceramide (LacCer) (Figure 1(f)) was examined in HUVECs and HAECs by RNAi-mediated silencing of LacCer synthase expression (GalT-V) and by use of the pharmacological inhibitor of LacCer synthase, d-threo-1-phenyl-2-decanoylamino-3-morpholino-1-propanol (d-PDMP). LacCer contributes to VEGF-induced platelet EC adhesion molecule-1 (PECAM-1) expression and angiogenesis [15, 16]. This suggests that LacCer is important in VEGF-implicated angiogenesis associated with coronary heart disease, vascular complications in diabetes, inflammatory vascular diseases, and tumor metastases. The functional involvement of other GSLs in angiogenesis is also implied. Inhibition of ganglioside GM3 (Figure 1(g)) biosynthesis with the glucosyltransferase inhibitor, *N*-butyldeoxynojirimycin (NB-DNJ), increased the EC proliferation and the phosphorylation of VEGFR-2 and Akt. The effects of NB-DNJ were reversed by the addition of GM3 [17]. Thus, this report concludes that GM3 has antiangiogenic action in ECs and may possess therapeutic potential for reducing tumor angiogenesis. 

 Sulfoglucuronosyl paragloboside (SGPG) (Figure 1(h)), a minor GSL in ECs, is a ligand for L-selectin. It has been implicated in neuroinflammatory diseases such as the Guillain-Barré syndrome. Inflammatory cytokines such as TNF-*α* and IL-1*β* upregulate SGPG expression in human brain cerebromicrovascular ECs (SV-HCECs) by stimulating the gene expression of the P and S forms of glucuronosyltransferases (GlcATp and GlcATs) and the human natural killer antigen (HNK-1) sulfotransferase (HNK-1 ST) [18]. Transfection of the SV-HCEC line with HNK-1 ST siRNA downregulated SGPG expression, inhibited cytokine-stimulated T-cell adhesion, and offered protection against apoptosis [19]. This paper indicates the functional importance of SGPG expression for brain-associated ECs in neuroinflammatory diseases. Recently, the SGPG cell signaling pathways were investigated. SGPG expression was inhibited by transfecting the cells with HNK-1 ST gene siRNA, and SGPG synthesis was promoted by overexpressing GlcATp and GlcATs. And then, either up- or downregulation of SGPG reduces activation of the NF-*κ*B pathway, which is mediated by the accumulation of inhibitor of *κ*B (I*κ*B) [20]. 

## 4. Proteoglycans

 Proteoglycans (PGs) are macromolecules composed of a specific core protein substituted with covalently linked glycosaminoglycan (GAG) chains, namely, chondroitin sulfate (CS), dermatan sulfate (DS), and heparan sulfate (HS). Hyaluronan (HA) is the only GAG synthesized in a free form not covalently bound to a core protein. GAGs are linear, negatively charged polysaccharides comprised of repeating disaccharides of acetylated hexosamines (*N*-acetyl-galactosamine or *N*-acetyl-glucosamine) and mainly uronic acids (d-glucuronic acid or l-iduronic acid) sulfated at various positions. In HA, there are no chemical modifications such as sulfation and epimerization.

PGs can be classified into two main groups according to their localization: those extracellularly secreted and those associated with the cell surface. The secreted group consists of PGs involving large aggregating PGs, namely, hyalectans (e.g., versican), small leucine-rich PGs (SLRPs), and basement membrane PGs (e.g., perlecan). Cell-surface-associated PGs are divided into two main subfamilies: syndecans and glypicans [21, 22]. PGs perform numerous biological functions, act as structural components in tissue organization, and affect several cellular parameters, such as cell proliferation, adhesion, migration, and differentiation. PGs interact with growth factors and cytokines, as well as with growth factor receptors, and they are implicated in cell signaling.

### 4.1. GAGs

#### 4.1.1. Heparan Sulfate (HS)

To clarify the roles of HS in endothelium under pathological conditions, HS expression on human ECs was studied following stimulation by inflammatory or hyperglycemic conditions. Inflammation is pivotal in atherosclerosis, and a key early step is endothelial dysfunction. It was found that IL-1, TNF-*α*, and IFN-*γ* influence HS expression significantly in HUVECs [23]. Also, C-reactive protein, the prototypic marker of inflammation and cardiovascular risk marker, has been shown to promote atherogenesis, and increased levels of C-reactive protein are associated with endothelial dysfunction. C-reactive protein treatment caused the expression of HS to significantly reduce [24]. In diabetes, the endothelium is exposed chronically or transiently to hyperglycemic conditions. In addition, endothelial dysfunction in diabetes is related to changes in the inflammatory response and turnover of the extracellular matrix. In hyperglycemic conditions, short-term inflammatory stimuli affected both the size and the sulfation pattern of HS on ECs, with the outcome depending on the type of stimulus [25]. Thus, modification of HS under pathological conditions is considered a major cause of endothelial dysfunction, resulting in the disturbance of vascular integrity and barrier properties, due to decreased negative charge and increased permeability, and the consequent release of bioactive substances such as cytokines, enzymes, and growth factors.

 To date, several studies have investigated the binding capabilities of HS on human ECs. Adhesion of leukocyte to ECs is partly dependent on HS that binds to L-selectin [26]. The S100 family heterodimer, MRP-8/14 complex, which is abundantly expressed in inflamed endothelium, binds to ECs via the MRP-14 subunit, interacting chiefly with HS [27]. It has also been shown that HMGB1 (high-mobility-group protein B1), an inflammatory cytokine, and receptor for advanced glycation endproducts (RAGEs) bind to HS [28]. In another study, *P. falciparum*-infected erythrocytes (parasitized red blood cells) adhere to HS on ECs [29]. Binding via HS followed by sequestration may be related to the severity of the diseases. 

 Functional studies of HS on human ECs have been performed. In HUVECs, RNAi-mediated downregulation of HSulf-1, which selectively removes 6-*O*-sulfate from HS, resulted in increased proliferation mediated by HS-dependent FGF-2, hepatocyte growth factor, and VEGF165. HSulf-1 downregulation also enhanced downstream signaling through the extracellular signal-regulated kinase pathway, when compared with untreated cells [30]. Another study confirmed the importance of HS 6-*O*-sulfate in EC function [31]. Reducing HS 6-*O*-sulfotransferase-1 (HS6ST-1) or 6-*O*-sulfotransferase-2 (HS6ST-2) expression in ECs with RNAi affected the prevalence of HS 6-*O*-sulfate moieties in HS sequences; 1%–40% reduction in 6-*O*-sulfates significantly compromised FGF-2- and VEGF165-induced EC sprouting and tube formation. These data indicate that 6-*O*-sulfate moieties in endothelial HS are of major importance in regulating FGF2- and VEGF165-dependent EC functions.

#### 4.1.2. Chondroitin Sulfate (CS)

CD97, which is highly expressed on various inflammatory cells and some carcinomas, contributes to inflammation-mediated angiogenesis and possibly tumor progression. CD97 acts as a potent chemoattractant for the migration and invasion of ECs, and this function is integrin dependent. CD97 EGF-like repeat 4 is known to bind CS. One study [32] showed that coengagement of *α*5*β*1 and CS proteoglycan by CD97 synergistically initiates EC invasion.

#### 4.1.3. Hyaluronan (HA)

Chronic inflammation has a critical role in the onset of several diseases, including atherosclerosis, and endothelial dysfunction is a key early step in these diseases. A study [33] has shown that the expression of HA on HUVECs is induced by IL-15, which has been suggested to play a role in the setting of the chronic autoimmune disease, particularly in the recruitment and activation of synovial T-cells. The results of the study suggest that IL-15 can regulate EC function and thereby enable a CD44-initiated adhesion pathway that facilitates the entry of activated T lymphocytes into inflammatory sites. In HAECs, C-reactive protein, the expression of which is related to inflammation as well as endothelial dysfunction, dose dependently increased HA release. This is thought to result in EC dysfunction by, for example, increasing monocyte-EC adhesion [24]. Another study found that IL-1*β*, TNF-*α*, and TNF-*β* strongly induce HA synthesis via the NF-*κ*B pathway. Moreover, it has been verified that U937 monocyte adhesion to stimulated ECs depends strongly on HA [34]. Thus, in chronic inflammation, elevation of HA expression via inflammatory stimulation promotes adhesion of leucocytes to ECs, resulting in vascular-related diseases, such as atherosclerosis.

 Recent *in vivo* studies revealed that the inner blood vessel surface is lined with an endothelial surface layer at least 0.5 *μ*m thick, which serves as a shield protecting the vessel wall from arteriosclerosis. HA seems to be an essential component that is related to the atheroprotective properties of this surface structure. It has also been shown that HA is increased in a shear-stress-dependent manner via the phosphatidylinositol 3-kinase-Akt pathway [35, 36]. In particular, pulsatile, arterial-like shear stress conditions effectively induced HA [36]. Thus, fluid shear stress stimulates the incorporation of HA into the glycocalyx, which may contribute to its vasculoprotective effects against proinflammatory and pro-atherosclerotic stimuli. 

### 4.2. Extracellularly Secreted PGs

#### 4.2.1. Perlecan

It has been reported that the amounts of heparan sulfate (HS) in perlecan (an HSPG) are different in HUVECs and HAECs and that HS expression affects perlecan-dependent adhesion of vascular cells [37]. The functions of HS in endothelial perlecan were further investigated [38, 39]. The studies show that the binding abilities of FGF-1 and FGF-2 to endothelial perlecan differ depending on the HS structures in the different cell types. 

 Hyperglycemia is an independent risk factor for diabetes-associated cardiovascular disease. One potential mechanism involves hyperglycemia-induced changes in arterial wall extracellular matrix components leading to increased atherosclerosis susceptibility. A decrease in HS GAG has been reported in the arteries of diabetics. In HAECs, high glucose induced modification in HS but not in perlecan core protein levels [40]. In addition, endothelial dysfunction in diabetes is related to changes in the inflammatory response and the turnover of extracellular matrix. In hyperglycemic conditions, secretion of perlecan from ECs was increased after IL-1*β* stimulation [25].

 Perlecan, but not other HSPGs, is dramatically downregulated in ECs treated with antiangiogenic cleaved and latent forms of antithrombin [41, 42]. The previously established key role of perlecan in mediating FGF-2 stimulation of EC proliferation and angiogenesis suggests that a primary mechanism by which antiangiogenic antithrombins exert their effects is through the downregulation of perlecan expression. The role of perlecan in the antiangiogenesis function of NK4, an angiogenesis inhibitor, was also studied [43]. In this study, knockdown of perlecan expression in ECs by RNAi significantly reduced the inhibitory effect of NK4 on fibronectin assembly and cell spreading. Thus, this study indicates that the association of NK4 with perlecan plays a key role in angiogenesis inhibition by NK4.

#### 4.2.2. Endocan

Endocan is a novel small soluble dermatan sulfate proteoglycan (DSPG) produced specially by ECs. In SV40-transfected human ECs (SV1 cells), endocan regulates HGF/SF-mediated mitogenic activity and may support the function of HGF/SF, not only in embryogenesis and tissue repair after injury but also in tumor progression [44]. Additionally, endocan expression in HUVECs is upregulated by tumor-cell-conditioned medium [45]. Moreover, treatment with VEGF resulted in dose- and time-dependent increases in endocan mRNA. The results suggest that endocan is preferentially expressed in tumor endothelium *in vivo* and that its expression is regulated by tumor-derived factors. Now, it is highlighted that endocan is a marker of EC activation during growth of the new vessels required for tumor progression [46].

#### 4.2.3. Decorin

Decorin, a member of the small leucine-rich repeat proteoglycan (SLRP) family, is expressed by sprouting ECs during inflammation-induced angiogenesis *in vivo* and by human ECs cocultured with fibroblasts in a collagen lattice. Activation with IL-10 or IL-6 treatment induces decorin mRNA in human ECs [47]. As function of decorin in human ECs, it has been reported that decorin core protein can bind to and activate insulin-like growth factor-I receptor (IGF-IR) [48] and that decorin promotes *α*2*β*1 integrin-dependent EC adhesion and migration on fibrillar collagen type I [49]. It is now understood that modulation of cell-matrix interactions by decorin plays a key role during angiogenesis [50]. 

#### 4.2.4. Versican

Primary human ECs, if stimulated with TNF-*α* or VEGF, alter their expression of versican (a large aggregating CSPG) by *de novo* transcription of the V3 isoform and by exhibiting a moderate V1/V2 production. Induced versican synthesis and *de novo* V3 expression were also observed in ECs induced to migrate in a wound-healing model *in vitro* and in angiogenic ECs forming tubule-like structures in Matrigel or fibrin clots. Thus, in activated conditions, versican expression in human ECs is altered [51]. This study indicates that versican produced from ECs plays a key role in the pathological conditions such as inflammation, angiogenesis, and wound healing.

#### 4.2.5. Biglycan and PG-100

When EA.hy 926 cells, one of human ECs, form monolayer cultures typical of macrovascular ECs, they express and synthesize detectable amounts of biglycan and PG-100 (members of the small leucine-rich repeat proteoglycan family). TNF-*α*, responsible for changing the morphology of the cells from a polygonal to a spindle shape and for stimulating the detachment of the cells from the culture dish, markedly decreased the synthesis of biglycan. By contrast, PG-100 expression was increased in response to FGF-2, FGF-7, TNF-*α*, and TGF-*β* [52]. Although the functional roles of biglycan and PG-100 are not yet clearly understood, their different responses to the stimuli may be critical for the progression of vascular diseases. Another study has shown that antiangiogenic antithrombin treatment significantly decreases biglycan in HUVECs [42], suggesting that a mechanism of antiangiogenic antithrombins is through the downregulation of proangiogenic biglycan.

### 4.3. Cell Surface PGs

#### 4.3.1. Glypicans

Glypican-1 is the only glypican expressed in the vascular system. VEGF165 interacts with EC-derived glypican-1 dependent on HS [53]. Recently, the contribution of glypican-1 to the cell cycle and proliferation has been demonstrated in ECs [54].

#### 4.3.2. Syndecans

Proteoglycans (PGs) are important constituents of the plasma membrane and of the basement membrane supporting the EC layer. Changes in the amounts or the structures of PGs in the endothelium may affect important functions, such as turnover of lipoproteins, filtration properties, and regulation of chemokines during inflammation, which are all relevant to diabetes. In HUVECs, exposure to high glucose (hyperglycemic condition) leads to decreased secretion of syndecan-1 [55]. Thrombospondin-1 (TSP-1), an extracellular matrix protein, modulates focal adhesion in mammalian cells and exhibits dual roles in angiogenesis. There are indications that binding of TSP-1 to syndecan-4 proteoglycan mediates tubulogenesis and their protection from apoptosis [56]. Syndecan-2, the major syndecan expressed by human microvascular ECs (HMECs), is regulated by growth factors and extracellular matrix proteins, in both bidimensional and tridimensional culture conditions [57]. Downregulation of syndecan-2 reduced the spreading and adhesion of HMECs, and it not only enhanced their migration but also impaired the formation of capillary-like structures. Therefore, syndecan-2 has an important function in some of the necessary steps in the angiogenic process. Syndecan-1 is a critical regulator of *α*v*β*3 and *α*v*β*5 integrins during angiogenesis and tumorigenesis, and it is inhibited by the novel peptide called synstatin [58].

## 5. Glycoconjugate-Related Molecules: Galectins

Galectins are a family of lectins that bind to *β*-galactosides via a carbohydrate recognition domain containing many conserved sequence elements [59]. There are currently 15 known mammalian galectins [60], which are involved in a variety of biological processes [61]. Expression of galectin-1 in cultured human ECs was first shown in 1995 by Baum et al. [62]. They also showed that activation of cultured ECs by minimally oxidized low-density lipoprotein (MM-LDL) or cytokines and LPS increases galectin-1 expression, as determined by ELISA, northern blot analysis, and high-throughput cDNA sequencing. Another study found that poly IC, a synthetic analog of double-stranded RNA (dsRNA), enhances the expression of galectin-9 mRNA and protein in ECs in concentration- and time-dependent manners [63]. Treatment of cells with dsRNA *in vitro* mimics viral infection and regulates the expression of various genes. Thus, it has been proposed that upregulation of galectin-9 expression by poly IC in the vascular endothelium may be part of the mechanism for leukocyte trafficking through the vascular wall after viral infection. Subsequently, galectin-1, -3, -8, and -9 expression levels in quiescent ECs were measured, and the expression and distribution of these galectins changed after activation with tumor-derived culture medium [64]. Recently, it was reported that galectin-9 protein expression is positively regulated by histone deacetylase 3 in ECs [65]. This study provides new evidence that HDAC3 regulates galectin-9 expression in ECs via interaction with the PI3K-IRF3 signaling pathway.

 Some functional analyses of galectins in human ECs have been reported. Cancer-associated carbohydrate T antigen plays a leading role in docking breast and prostate cancer cells onto endothelium by specifically interacting with EC-expressed galectin-3 [66]. One of the low-molecular-weight synthetic lactulose amines (SLAs), *N*, *N*′-dilactulose-octamethylenediamine (D-LDO), severely impaired tube formation of ECs. This inhibitory effect is thought to occur by inhibition of galectin-1- and/or galectin-3-mediated functions. D-LDO inhibited the binding of galectin-1 and/or galectin-3 to the highly glycosylated protein 90 K. Thus, D-LDO may be a new galectin inhibitor for blocking angiogenesis [67]. In HUVECs, knockdown of galectin-3 and Mgat5, an enzyme that synthesizes high-affinity glycan ligands of galectin-3, reduced VEGF-A-mediated angiogenesis *in vitro*. A direct interaction was detected on the plasma membrane between galectin-3 and VEGF-R2, and this interaction was dependent on the expression of Mgat5. Using immunofluorescence and cell surface labeling, an increase in the level of internalized VEGF-R2 was observed in both Mgat5 and galectin-3 knockdown cells, suggesting that galectin-3 retains the receptor on the plasma membrane via lattice formation. Thus, galectin-3 contributes to the plasma membrane retention and proangiogenic functions of VEGF-R2 in ECs [68].

## 6. Glycan-Degrading Enzymes

### 6.1. Heparanase

Heparanase is an endo-*β*-d-glucuronidase responsible for heparan sulfate (HS) degradation at a limited number of sites, yielding HS fragments of an appreciable size (5–7 kDa) and with biological potency [69, 70]. In 1991, heparanase activity was observed in human ECs [71]. Few reports of functional analyses of endogenous heparanase have since been published. The HS content of the endothelium is reduced under hyperglycemic conditions, and it may contribute to the pathogenesis of atherosclerosis. This suggests that HS reduction in ECs is due to increased heparanase production under hyperglycemic conditions. Recently, high-glucose-induced heparanase production and HS degradation were detected in human several types of ECs [72, 73]. In fact, functional analyses revealed that the expression of heparanase contributed to EC migration and proliferation. Furthermore, it has been shown that EC proliferation and migration correlate with Akt and ERK phosphorylation levels [72].

### 6.2. Sialidase

Sialidases are glycosidases that catalyze the removal of *α*-glycosidically linked sialic acid residues from the carbohydrate groups of glycoproteins and glycolipids. To date, at least 4 mammalian sialidases, NEU1–4, have been identified [74, 75]. Whether the EC surface expresses sialidase activity was not known until recently. In human lung microvascular ECs (HMVEC-Ls) and human pulmonary artery ECs (HPAECs), NEU1–4 expression was examined, and functional analysis by knockdown and overexpression was performed. These ECs express catalytically active NEU1 and NEU3 sialidases, and NEU1 restrains the endothelial migratory response to wounding. It was concluded that NEU1 regulates endothelial remodeling in response to injury [76].

## 7. Closing Remark

**Figure 2:**
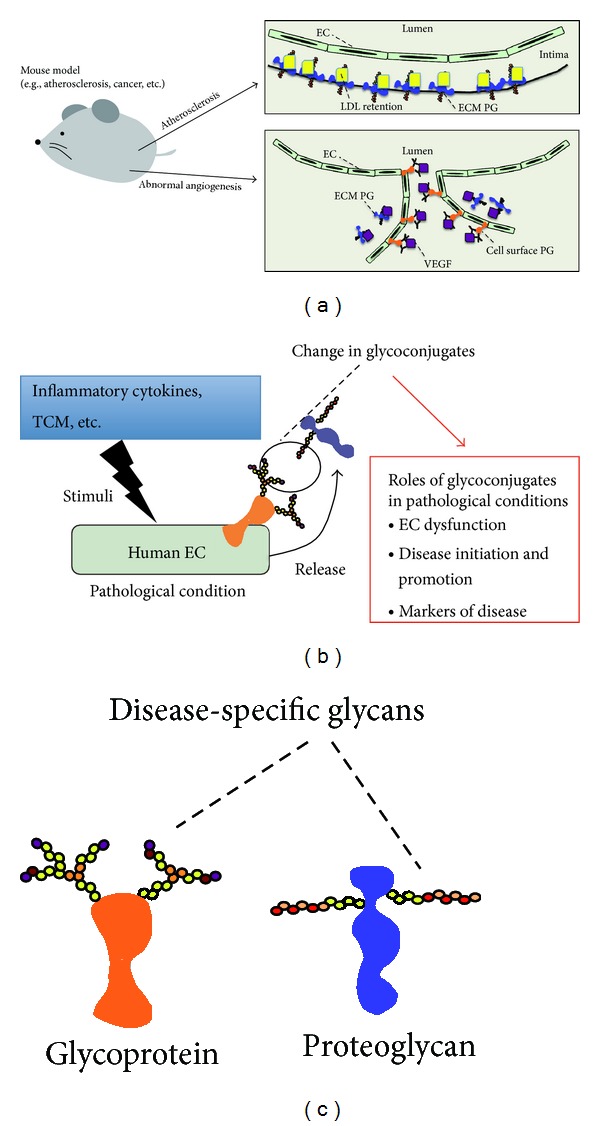
Glycoconjugates and peripheral artery disease (PAD). (a) The contribution of glycoconjugates to vascular-related diseases has been examined in mouse models. Initiation and promotion of atherosclerosis via interaction between LDL and proteoglycans (PGs) have been indicated. Additionally, the importance of the interaction between growth factors (e.g., VEGF165) and heparan sulfate PG in ECs and extracellular matrix (ECM) during abnormal angiogenesis, such as in tumorigenesis, has been shown. (b) In human ECs, pathological stimulation, such as inflammatory cytokines and tumor-cell-derived medium (TCM), induces changes in glycoconjugates (e.g., expression levels, glycan structures, etc.). These changes may lead to EC dysfunction and disease initiation and promotion. Furthermore, glycoconjugates specifically modified under pathological conditions may be candidates for markers of PAD. (c) Study of human ECs to identify specific glycoconjugates related to PAD may be a good strategy for the prevention and treatment of PAD.

In this paper, we described previous research about glycoconjugates in human ECs. To date, the functional roles of glycoconjugates *in vivo* have been investigated in animal disease models (e.g., atherosclerosis) (Figure 2(a)). In apoE-null mice, HS of perlecan contributes to the promotion of atherosclerosis [77]. This contribution may involve increased LDL retention, altered vascular permeability, or other mechanisms. Additionally, a proatherosclerosis function of biglycan has been suggested by the retention of LDL [78]. Abnormal angiogenesis plays a critical role in the pathogenesis of many diseases such as cancer, ischemic vascular disorders, and diabetic retinopathy [79]. In particular, the importance of HS in tumor angiogenesis has been demonstrated by tumor-based studies in mice with endothelial-targeted genetic alterations in HS biosynthesis (Figure 2(a)) [80]. Thus, the functional importance of glycoconjugates in vascular-related diseases has been demonstrated.

 Glycoconjugates are markers for cell context, and they play biological roles. Increasing evidence from examination of human ECs indicates that glycoconjugates also serve as biomarkers and functional players in the human endothelium under pathological conditions as described here (Figure 2(b)). Therefore, although many issues await clarification, glycoconjugates are attractive potential targets for the prevention and treatment of vascular-related diseases. In recent years, the incidence of vascular-related diseases, such as cancer, cardiac disease, and cerebrovascular disease, has increased with an aging population, and prompt countermeasures are sought. Hence, further study of the functional roles of glycoconjugates in human ECs and identification of specific glycoconjugates related to disease are required in order to develop therapeutic strategies for vascular-related diseases, such as peripheral artery disease (PAD) (Figure 2(c)).
